# Quality of Life, Depression, and Anxiety in Survivors of Critical Illness from a Greek ICU. A Prospective Observational Study

**DOI:** 10.3390/healthcare9070849

**Published:** 2021-07-05

**Authors:** Charikleia S. Vrettou, Vassiliki Mantziou, Ioannis Ilias, Alice G. Vassiliou, Stylianos E. Orfanos, Anastasia Kotanidou, Ioanna Dimopoulou

**Affiliations:** 1 First Department of Critical Care Medicine & Pulmonary Services, School of Medicine, Evangelismos Hospital, National and Kapodistrian University of Athens, 106 76 Athens, Greece; mantziouv@yahoo.gr (V.M.); alvass75@gmail.com (A.G.V.); stylianosorfanosuoa@gmail.com (S.E.O.); akotanid@gmail.com (A.K.); 2 Department of Endocrinology, Diabetes & Metabolism, Elena Venizelou Hospital, 115 21 Athens, Greece; iiliasmd@yahoo.com

**Keywords:** quality of life, critical illness, anxiety, depression, post intensive care syndrome

## Abstract

The physical and psychological consequences of critical illness may affect intensive care unit survivors for up to five years, and hence, health-related quality of life has emerged as an important measure of outcome in this population. We aimed at investigating the quality of life, the presence of anxiety and depression symptoms, and the correlations of clinical and psychological parameters with the quality of life scores in survivors of critical illness one year after discharge from intensive care. Widely used scales that have been validated for the Greek population were used. One thousand two hundred and seventy (1270) patients were assessed for eligibility. Inclusion criteria were age between 18 and 68 years and the presence of critical illness requiring endotracheal intubation and mechanical ventilation for more than three days. One hundred and four (104) patients were included in the final analysis; forty-nine age and sex-matched healthy volunteers were included for comparison. One year following intensive care unit discharge, survivors of critical illness had impaired quality of life scores, as measured with the WHOQOL-Bref, compared to healthy subjects (*p* < 0.001 for the physical and psychological domains, and *p* = 0.006 for the domain of social relationships). In addition, we found that quality of life scores were significantly lower in patients with comorbidities (*p* < 0.01), and correlated with the duration of ICU stay (r = −0.19, *p* = 0.04) and with the presence of symptoms suggestive of depression (r = −0.66, *p* < 0.0001) and post-traumatic stress disorder (r = −0.61, *p* < 0.0001). Approximately one-third of our patients scored high in the CES-D scale for depression, while the majority of them scored high in the STAI scale for anxiety symptoms.

## 1. Introduction

Technological advances in intensive care have gradually reduced intensive care unit (ICU) mortality; the consequences, however, of critical illness affect the physical, cognitive, and mental health of ICU survivors. These consequences are recognized as a separate pathophysiological entity, entitled post-intensive care syndrome (PICS), and may have a strong, negative impact on functioning and health-related quality of life (HRQOL). For these reasons, HRQOL has emerged as an important measure of outcome in ICU survivors. Numerous studies, including meta-analyses, have reported poor HRQOL in ICU survivors in the months and years following hospital discharge when compared to normal controls [1,2,3]. Factors related to worse HRQOL are age, sex, health status prior to ICU admission, length of ICU stay, the severity of illness, the presence of organ failures, the admission diagnosis, and the quality of sleep after ICU discharge [1,2,3,4,5,6].

Even though HRQOL scores are increasingly used in studies and are nowadays recognized as important outcome parameters, such data may be difficult to interpret or compare, mostly due to the diversity of the methodologies used. More specifically, the time required for HRQOL recovery is not defined, and therefore, the optimal follow-up period for evaluation remains undetermined. The instruments used in the studies to assess HRQOL, along with other psychometric tools, are different, thereby complicating the systematization and interpretation of results. The populations studied may also differ, ranging from general ICU patients to patients with respiratory failure or trauma [7,8]. Finally, the parameters in each study can also vary significantly. Some studies have focused on factors that existed prior to critical illness, others on the severity of critical illness and ICU-related factors, while others have focused mostly on post-ICU factors and related interventions [9].

We designed a prospective observational study that aimed to investigate HRQOL, the presence of depressive symptoms and anxiety in patients discharged from a general ICU. We additionally aimed at investigating the correlations between clinical and social parameters and HRQOL. To this end, we applied commonly used scales for the symptoms of depression and anxiety, as well as for HRQOL assessment, which have been validated for the Greek population. We hypothesized that HRQOL is compromised in ICU survivors and that depression and anxiety symptoms are also present.

## 2. Materials and Methods

### 2.1. Patients

The study was performed in the ICU of Evangelismos General Hospital in Athens, Greece, a 45-bed general ICU that admits unselected medical and surgical cases, including trauma. All patients admitted to the ICU from 1 August 2017 through 31 December 2019 were assessed for eligibility. We evaluated ICU survivors one year after ICU discharge. Inclusion criteria were age between 18 and 68 years, ICU admission requiring endotracheal intubation and mechanical ventilation for more than three days, and survival at the time of the interview. We restricted inclusion age below 68 years since this is the usual retirement age in Greece, and we wanted to assess the “return to previous occupation” as a component of the quality of life. Exclusion criteria included the inability to attend the interview due to a long distance of residence, the inability to communicate, or still in a rehabilitation facility. Eligible patients were assessed for participation by telephone contact, and if patients agreed, an appointment for psychometric assessment was scheduled. Data retrieved from the electronic medical records included patients’ demographics, the reason for hospital and ICU admission, length of ICU stay, previous medical history, and comorbidities. Additional information collected during the interview included family status, educational status, and occupation information. During the appointments, the patients were asked to complete the relevant questionnaires. All interviews were performed by a trained psychologist. Since there are no normal cut-off values for the WHOQOL-Bref, the questionnaire was also administered to a control group of subjects, age and sex-matched, who had never been admitted to the ICU and had no known comorbidities.

The ethics committee of the Hospital approved the study (21 June 2018, No 220). A written informed consent was obtained from all study participants.

### 2.2. Questionnaires

#### 2.2.1. The WHOQOL-Bref Questionnaire—Assessment of the Quality of Life

The World Health Organization Quality of Life (WHOQOL)-Bref is a self-administered questionnaire designed to assess the HRQOL of individuals over the preceding two weeks. It is a shorter version of the WHOQOL-100. Both were developed by WHO and were published in 1995. The WHOQOL-Bref questionnaire is one of the best-known instruments that has been developed for cross-cultural comparisons of quality of life, and it is available in many languages and has been validated for the Greek population [10]. It is composed of 26 questions on the individual’s perceptions of health and well-being and covers four domains: physical health, psychological health, social relationships, and environmental quality of life. Responses to questions are on a 1–5 Likert scale. Raw domain scores are then transformed to a 1–100 score according to an equation provided in the instructions. Formal training is recommended for scoring and interpretation purposes; however, minimal training is required for administration [11]. There are no normal cut-off values for the WHOQOL-Bref, and therefore, the questionnaire was administered to a control group of healthy subjects, matched with our patients for age and sex.

#### 2.2.2. The State-Trait Anxiety Inventory (STAI)

The STAI is a 40-item questionnaire assessing two types of anxiety: state (a temporary assessment of the level of anxiety someone is currently experiencing) and trait (a predisposition to anxiety). The scale has been validated for the Greek population [12]. The questionnaire is structured on 4-point Likert scales (from 1 = not at all, to 4 = extremely). The scores for each subscale are added and can range between 20 and 80, with a higher score indicating worse anxiety. Normative data are available for working adults, college students, high school students, and military recruits. A cutoff score of 40 is commonly used to define probable clinical levels of anxiety for both state and trait subscales [13].

#### 2.2.3. The Center for Epidemiologic Studies Depression (CES-D) Scale

The Center for Epidemiologic Studies Depression (CES-D) Scale was developed specifically for research use in the general (i.e., non-psychiatric) population. This self-report instrument consists of 20 items. For each item, respondents indicate the frequency or duration for a specific feature during the preceding week (ranging from 0 = rarely or none of the time to 3 = more or all of the time). Sixteen items tap a variety of cognitive, affective, behavioral, and somatic symptoms associated with depression; four focus on positive moods, scored in reverse direction (i.e., a raw score of 0 = 3, 1 = 2, 2 = 1, and 3 = 0). Total scores range from 0 to 60, with higher scores indicating greater distress [14]. It has been validated for the Greek population with high sensitivity and specificity at the cutoff level 24 [15].

#### 2.2.4. The Impact of Event Scale-Revised (IES-R)

The IES-R was designed as a measure of post-traumatic stress disorder (PTSD) symptoms and is a short, easily administered self-report questionnaire. The scale has been translated to Greek and validated for the Greek population [16]. The IES-R is a 22-item scale [17]. Responders are asked to report how distressed or bothered they are over the past 7 days by symptoms related to a specific trauma, using the following scale: “not at all” (item score 0), “a little bit” (score, 1), “moderately” (score, 2), “quite a bit” (score, 3), or “extremely” (score, 4). There are three subscales: intrusion (intrusive thoughts, nightmares, intrusive feelings and imagery, dissociative-like re-experiencing), avoidance (numbing of responsiveness, avoidance of feelings, situations, and ideas), and hyperarousal (anger, irritability, hypervigilance, difficulty concentrating, heightened startle). A total score is calculated, ranging from 0–88. A score of 33 and above represents the best cutoff for a probable diagnosis of PTSD.

#### 2.2.5. The Mini-Mental State Exam (MMSE)

The Mini-Mental State Examination (MMSE), or Folstein test, is extensively used in clinical and research settings to measure cognitive impairment. The test has been translated to Greek and validated for the Greek population [18]. The MMSE consists of a variety of questions, and it has a maximum score of 30 points and can be completed in five to ten minutes. The questions are grouped into seven categories, each representing a different cognitive domain or function: orientation, registration (repeating named prompts), attention, calculation, recall, language, and ability to follow simple commands. It was originally introduced in order to differentiate organic from functional dementia in psychiatric patients [19], while the Cochrane group found it to be a valid screening tool for dementia. The test was created based on an educational level of eight years. When using a cut-off value of 24, it has a sensitivity of 85% and a specificity of 90% for the diagnosis of dementia. The trend has been to classify the degree of cognitive impairment into three levels: 24–30 = no cognitive impairment; 18–23 = mild cognitive impairment; and 0–17 = severe cognitive impairment.

### 2.3. Statistical Analysis

Kolmogorov–Smirnoff tests were performed to test for normality. Descriptives are presented as mean ± standard deviation (SD) for variables with normal distribution or median (interquartile range, IQR) for variables with skewed distribution. Non-parametric Mann–Whitney U tests were used for comparisons between patients’ groups and for comparisons between patients and healthy controls. Correlations were performed by Spearman’s correlation coefficient. All aforementioned analyses were performed using the IBM SPSS statistics 26 software (SPSS, Chicago, IL, USA; version 26.0). The significance level was *p* < 0.05 for all tests used.

## 3. Results

### 3.1. Patients’ Characteristics

**Figure 1:**
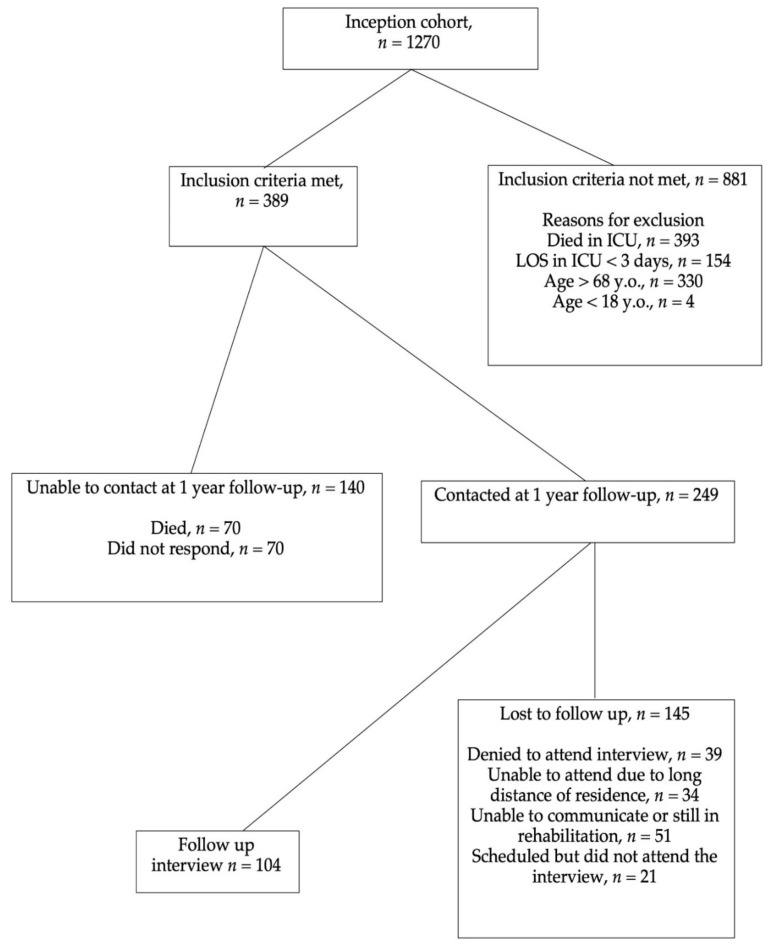
Study Flowchart.

During the study period, 1270 patients were admitted to the ICU. Figure 1 describes the study flowchart and patient follow-up. Of these, 104 patients (41% female) were included in the present study. The mean age of our cohort was 46 ± 14 years. Patients’ characteristics, including demographics, diagnosis in the ICU, comorbidities, ICU length of stay, education level, family status, the status of living, employment, and self-rated health status, are presented in Table 1.

A group of 49 control subjects consisting of 29 males and 20 females with a mean age of 43 ± 13 years was also included. Control subjects had never been admitted to an ICU and had no known comorbidities.

### 3.2. WHOQOL-Bref Questionnaire in ICU Survivors and Healthy Controls

**Figure 2:**
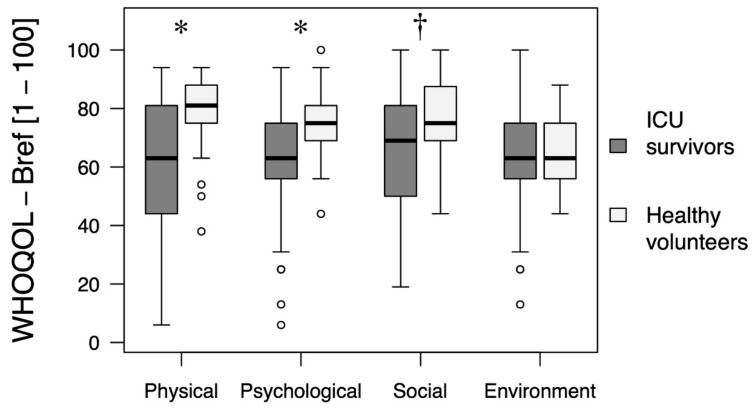
A comparison of the quality of life between patients discharged from the Intensive Care Unit and the group of healthy volunteers, as assessed by the WHOQOL-Bref questionnaire. * *p*-value < 0.001; ^†^ *p*-value = 0.006.

The results of the four domains of the questionnaire, including physical and psychological health, social relationships, and environmental quality of life, in ICU survivors and the control group are presented in Figure 2. As shown, ICU survivors had statistically significantly lower scores in the domains of physical and psychological health as well as in social relationships compared to the control group. The environmental quality of life was similar in the two groups.

### 3.3. Psychometric Characteristics in ICU Survivors

The scores for the two anxiety scales (S-STAI, *n* = 104; T-STAI *n* = 81), the CES-D for depression (*n* = 104), the IES-R for assessing post-traumatic stress disorder (*n* = 60), and the MMSE for assessing cognitive impairment (*n* = 104) are shown in Table 2. Not all patients managed to complete all the questionnaires administered. Since our primary aim was the assessment of the quality of life, all patients who completed the WHOQOL-Bref questionnaire were included. The most prominent abnormality was anxiety, affecting a large percentage of ICU survivors. PTSD and depressive symptoms were present in about one-third of the cohort. Cognitive impairment was less common.

### 3.4. Factors Related to the Scores in the Different Domains of the WHOQOL-Bref

The scores in the four domains of the WHOQOL-Bref questionnaire exhibited positive correlations. More specifically, the physical domain score correlated with the scores of the psychological (r = 0.68, *p* < 0.0001), social (r = 0.59, *p* < 0.0001), and the environmental quality-of-life (r = 0.51, *p* < 0.0001) domains. Patients with comorbidities had lower scores in all domains compared to patients with no comorbidities; physical (56 vs. 64, *p* < 0.0001), psychological (56 vs. 72, *p* < 0.0001), social (69 vs. 75, *p* < 0.0001), and environmental (63 vs. 75, *p* = 0.01). ICU stay duration correlated negatively with the physical (r = −0.19, *p* = 0.04) and the social relationship (r = −0.19, *p* = 0.04) domains. In contrast, there were no differences in the HRQOL between patients of different sex, ICU admission diagnosis (surgical and trauma vs. medical patients), education level (high school vs. higher degree), or living conditions (alone or with others).

Negative correlations were established between the physical domain component of the WHOQOL-Bref score and the CES-D scale for depression (r = −0.66, *p* < 0.0001) and the IES-R scale for PTSD (r = −0.61, *p* < 0.0001). Similar significant negative correlations were found between the CES-D and the IES-R with the other domain scores (psychological, social relationships and environmental quality of life) of the WHOQOL-Bref.

## 4. Discussion

In the present prospective study, we showed that ICU survivors had impaired HRQOL scores compared to age and sex-matched healthy subjects one year after ICU discharge. The factors related to HRQOL include the presence of comorbidities, the duration of ICU stay, and the presence of symptoms suggestive of depression and post-traumatic stress disorder. There were no differences in the HRQOL between patients of different sex, type of ICU admission (surgical and trauma vs. medical patients), level of education (high school vs. higher degree), and living conditions (living alone or with others). Approximately one-third of ICU survivors scored high in the CES-D scale for depression, as reported in previous studies [20]. The percentage of patients with abnormal anxiety scores in our sample was significantly higher than previously reported [21], reaching 72% and 96% for the S-STAI and the T-STAI scale, respectively.

Our results are in agreement with previous works supporting the significant role of comorbidities in quality of life post-ICU discharge; indeed, some authors even single out comorbidities as the most important risk factor for impaired HRQOL [9]. One possible explanation for this finding is that lack of comorbidities is compatible with better recovery of physical function. This also partly explains why rehabilitation trials testing numerous interventions during and after ICU have been proven ineffective in improving HRQOL [9]. There is, however, a counterargument in this theory. Orwelius et al. [22] used questionnaires administered to ICU patients who survived 6 months after ICU discharge to retrospectively assess pre-ICU health status. Their analysis suggested that even though the preexisting disease was a strong independent determinant of HRQOL, it was potentially subject to recall and response bias. In view of this argument, it is important that, in our study, results are based on medical records retrieved at the time of ICU admission rather than on post-ICU evaluation. Our results add to the body of evidence emphasizing the importance of considering comorbidities when discussing HRQOL as an outcome. We were not able to establish a relationship between impaired HRQOL and factors that have been previously related to worse HRQOL, such as unemployment, living alone, and the presence of cognitive decline [7,8]. It is worth mentioning, though, that in our population, the frequency of cognitive decline, as described by the MMSE, was only 11%. This significantly lower percentage compared to other studies [23] is not surprising due to the inclusion of a younger population.

Both the length of ICU stay and the presence of depressive symptomatology, as measured by the CES-D, were significantly related to HRQOL. Regarding the length of ICU stay, it has not been consistently reported to be related to worse HRQOL [1,2,3,8]. On the contrary, most studies examining depressive symptomatology state that post-ICU depressive symptoms were associated with substantially lower HRQOL [24]. A high score in the CES-D scale for depressive symptoms was observed in 28% of our study population, a frequency comparable to that described in the literature, ranging from 28% to 33% [24]. Early post-ICU depressive symptoms are identified as a strong risk factor for subsequent depressive symptoms in ICU survivors, and therefore, the mental health and well-being of this population can be affected for prolonged periods of time. Since the presence of depressive symptoms can significantly impact not only mental health but also physiological health and rehabilitation [25], further research is warranted in this area.

Symptoms attributed to PTSD also negatively correlated with all the domain scores of the WHOQOL-Bref. Almost one-third of the patients in our study scored higher than the cutoff value in the IES-R scale. Interestingly, PTSD was once believed to stem primarily from experiences such as combat, assault, and exposure to natural disasters. It is now accepted as the most frequently identified anxiety disorder in ICU survivors. Despite being repeatedly identified as a factor affecting HRQOL, there is still an ongoing debate on the prevalence and severity of PTSD in ICU survivors, and the reported PTSD prevalence rates in different studies range from 5% to 63%. In a recent meta-analysis, the one-year prevalence of PTSD was reported to be approximately 20% [26]. We observed a higher rate of reported PTSD symptoms. What is more striking, though, is the frequency of abnormally high scores in the S-STAI and T-STAI scales for state and trait anxiety symptoms, respectively. The majority of our patients scored high in both scales, 96% and 72% for the S-STAI and the T-STAI, respectively. In the literature, the frequency for nonspecific anxiety ranges from 23% to 41% [21]. There are at least two possible explanations for this deviation. On the one hand, a high percentage of the interviews took place during the COVID-19 pandemic. Thus, a higher anxiety level is reasonably expected, particularly for health-related matters [27]. On the other hand, our study population included younger patients, who frequently returned to previous activities and employment. These features can be related to high expectations that may stimulate anxiety for their fulfilment.

Our results could have implications for future research and planning of therapeutic interventions. We provided supporting evidence to the notion that comorbidities play an important role in HRQOL outcomes in ICU populations. Most of the therapeutic interventions after ICU discharge focus on improving functional ability by means of physiotherapy and rehabilitation, and for good reasons. Critical illness can have devastating consequences on functional ability, muscular strength, and physiological reserve. However, little is known about the effect of different treatments on symptoms of anxiety and depression after ICU discharge. Likewise, little is known about the psychological morbidities of those patients who manage to achieve a good functional recovery and even return to work. They may represent a part of the population willing and able to contribute, who could significantly benefit from appropriate interventions. The best course of action for these patients could be early intervention, even prior to ICU discharge [28]. Interestingly, it has been reported that the mental health domain scores initially improved during the first year following ICU discharge and then declined to the hospital discharge levels at two years [7]. Inadequate psychological intervention could be an explanation for the delayed improvement of HRQOL or even a lack thereof.

There are several limitations to our study. Firstly, our data did not contain information on the severity of illness, such as the Acute Physiology and Chronic Health Evaluation (APACHE II) or the Sequential Organ Failure Assessment (SOFA) scores. The inclusion of such data would have allowed us to add to the existing knowledge on the relative importance of preexisting comorbidities versus acute illness factors in relation to HRQOL [1,9]. Secondly, the lack of power analysis for sample size estimation and the high mortality and dropout rate in our initial population increased the risk of bias. Indeed, the final sample of our cohort included mostly patients with good recovery who were able to resume their previous activities. Nevertheless, the impact on their quality of life was significant, and their stress levels were higher than the levels described in other studies. A ratio different from the usual 1:1 between cases and control subjects was chosen because the search for participants in the cases group provided more subjects than the search for the control group. This would be expected to lead to loss of power, yet, for a significance level (alpha) of 0.05, a post hoc calculation regarding the comparisons of quality of life scales yielded a power level of 0.818. It would have been more impactful to compare the ICU survivor group to a chronic condition group who had never been admitted to an ICU. Moreover, our results are descriptive; no inference on the effect of tentative covariates was attempted because our sample could be considered to be one of convenience and not a random one. Finally, the sample size was relatively small, making it difficult to generalize our findings. Notwithstanding these limitations, this was a prospective study that applied different psychometric scales in order to assess the mental well-being of ICU survivors; it comprises one of the few studies reporting outcomes from southern Europe and the only one we are aware of in the Greek population.

## 5. Conclusions

In conclusion, our findings support that pre-existing comorbidities, the length of ICU stay, and the presence of depressive and PTSD-related symptoms are the main factors related to worse WHOQOL-Bref scores in critically ill patients one year after ICU discharge. High levels of anxiety may be present in ICU survivors who achieve a good recovery, and this finding warrants further investigation since this population could benefit significantly from appropriate psychological and medical support.

## Figures and Tables

**Table 1:** The study population’s clinical and social characteristics and self-rated health status.

Characteristics	Results
Age, years ^1^	46 ± 14
Gender (male) ^2^	63 (59%)
Diagnostic Category ^2^	
Medical	37 (36%)
Surgical Acute	23 (22%)
Surgical Elective	12 (12%)
Trauma	32 (31%)
Comorbidities	
At least one comorbidity present ^2^	69 (66%)
Cardiovascular	26
Respiratory	12
Neurologic	18
Oncology and Hematology	17
Autoimmune	9
Psychiatric	12
Diabetes	6
Chronic Renal Failure	6
ICU Length of Stay ^3^	11 (3–66)
Education ^2^	
<High school	20 (19%)
High school	31 (30%)
>High school	53 (51%)
Family status ^2^	
Single	40 (38%)
Married	48 (46%)
Estranged	4 (4%)
Divorced	10 (10%)
Widowed	2 (2%)
Living with others ^2^	
Yes	89 (86%)
No	15 (14%)
Employment ^2^	
Full time	50 (48%)
Part time	9 (9%)
Student	7 (7%)
Housekeeping	11 (10%)
Retired ^4^	9 (9%)
Unemployed	18 (17%)
Self-Rated Health Status ^2^	
Excellent	27 (26%)
Very Good	39 (37%)
Good	29 (28%)
Fair	7 (7%)
Poor	2 (2%)

ICU, Intensive Care Unit; ^1^ Mean ± Standard Deviation (SD); ^2^ Absolute number (%); ^3^ Median (Interquartile Range); ^4^ At the time of the interview.

**Table 2:** The follow-up assessment results for anxiety, depression, post-traumatic stress disorder, and cognitive function.

Evaluation Scale	Scores Median (Interquartile Range)	Percentage above/below Cutoff Value
The State-Trait Anxiety Inventory (STAI)		
S-STAI (*n* = 104)	50 (45–54)	100 (96%) ^a^
T-STAI (*n* = 81)	43 (39–47)	58 (72%) ^a^
The Center for Epidemiologic Studies–Depression (CES-D) scale (*n* = 104)	15 (8–27)	29 (28%) ^a^
The Impact of Event Scale—Revised (IES-R) (*n* = 60)	21 (4–40)	20 (33%) ^a^
The Mini-Mental State Exam (MMSE) (*n* = 104)	28 (26–29)	12 (11%) ^b^

^a^ Values higher than the accepted normal cutoff; ^b^ Value lower than the accepted normal cutoff; *n*, number of patients who completed the relevant questionnaire.

## Data Availability

Data related to the study are available by the authors upon reasonable request.
